# P02.14. Efficacy of ah shi point acupuncture on acne vulgaris

**DOI:** 10.1186/1472-6882-12-S1-P70

**Published:** 2012-06-12

**Authors:** B Son, Y Yun, I Choi

**Affiliations:** 1 Kyung Hee University Hospital at Gangdong, School of Oriental Medicine, Seoul, Republic of Korea

## Purpose

Ah shi point acupuncture involves inserting needles at painful or pathological sites. The purpose of this study is to evaluate the efficacy of ah shi point and general acupuncture point treatment of acne vulgaris.

## Methods

Thirty-six subjects were recruited and randomised in a double-blind (patient-blind and observer-blind) controlled trial to receive acupuncture either at general acupuncture points only or at both general acupuncture points and ah shi points 12 times over 6 weeks. The subjects were evaluated using the following outcome measurements: an inflammatory lesion count; a quality-of-life scale (Skindex-29); and a subjective symptom score.

## Results

After 12 treatment sessions, there was a significant reduction in the inflammatory acne lesion counts, the Skindex-29 scores and the subjective symptom scores from baseline in both groups, but no significant difference between groups.

## Conclusion

Acupuncture treatment of moderate acne vulgaris was associated with reduction of inflammatory lesions and improvement in quality of life.

